# The implications of the COVID-19 pandemic on eating disorder features and comorbid psychopathology among adolescents with anorexia nervosa and matched controls: a comparative cohort design study

**DOI:** 10.1007/s40519-024-01640-0

**Published:** 2024-02-12

**Authors:** Katrien Bracké, Cathelijne Steegers, Tess van der Harst, Rozemarijn Pons, Jeroen Legerstee, Bram Dierckx, Pieter de Nijs, Marieke Bax-van Berkel, Annemarie van Elburg, Marion Hekkelaan, Joke Hokke, Hetty de Jong-Zuidema, Lucas Korthals Altes, Farida Lengton-van der Spil, Judith Luijkx, Femke Schuurmans, Carien Smeets, Lia van Wijk, Claire Woltering, Meike Vernooij, Manon Hillegers, Tonya White, Gwen Dieleman

**Affiliations:** 1 Department of Child and Adolescent Psychiatry/Psychology, Erasmus MC-Sophia Children’s Hospital, P.O. Box 2060, 3015 GD Rotterdam, The Netherlands; 2 Department of Radiology and Nuclear Medicine, Erasmus MC, Rotterdam, The Netherlands; 3 Department of Pediatrics, The Van Weel-Bethesda Hospital, Dirksland, The Netherlands; 4 Altrecht-Rintveld, Mental Health Care Organisation for Eating Disorders, Zeist, The Netherlands; 5 Department of Pediatrics, Het Reinier de Graaf Gasthuis, Delft, The Netherlands; 6 Emergis-Ithaka, Mental Health Care Organisation for Child and Adolescent Psychiatry, Kloetinge, The Netherlands; 7 GGZ Delfland, Mental Health Care Organisation for Child and Adolescent Psychiatry, Delft, The Netherlands; 8 LUMC-Curium, Mental Health Care Organisation for Child and Adolescent Psychiatry, Leiden, The Netherlands; 9 Department for Eating Disorders, Emergis, Organisation for Mental Health and Well-Being, Goes, The Netherlands; 10 GGZ Westelijk Noord Brabant, Mental Health Care Organisation for Child and Adolescent Psychiatry, Roosendaal and Bergen Op Zoom, Bergen Op Zoom, The Netherlands; 11 Department of Pediatrics, The Bravis Hospital, Bergen Op Zoom, The Netherlands; 12 Department of Pediatrics, Elisabeth-TweeSteden Hospital, Tilburg, The Netherlands; 13 Department of Pediatrics, Franciscus Gasthuis en Vlietland, Rotterdam, The Netherlands; 14 Section on Social and Cognitive Developmental Neuroscience, National Institutes of Mental Health, Bethesda, MD USA

**Keywords:** Anorexia nervosa, COVID-19 pandemic, Treatment outcome, Depressive symptoms, Exercise

## Abstract

**Purpose:**

To examine implications of the COVID-19 pandemic on eating disorder (ED) features and psychopathology in female adolescents with anorexia nervosa (AN).

**Method:**

In total 79 females with first-onset AN (aged 12–22 years) were included and were followed up across a period of 1 year. We assessed AN participants recruited pre-pandemic (*n* = 49) to those recruited peri-pandemic (*n* = 30). Pre- (*n* = 37) and peri-pandemic (*n* = 38) age-, and education-matched typically developing (TD) girls (*n* = 75) were used as a reference cohort. ED features and psychopathology were assessed at baseline. After 1 year of follow-up the association between pandemic timing and clinical course was assessed. Analyses of covariance were used to examine differences in ED features and psychopathology.

**Results:**

Peri-pandemic AN participants experienced less ED symptoms at baseline compared to pre-pandemic AN participants. In particular, they were less dissatisfied with their body shape, and experienced less interpersonal insecurity. In addition, the peri-pandemic AN group met fewer DSM-IV criteria for comorbid disorders, especially anxiety disorders. In contrast, peri-pandemic AN participants had a smaller BMI increase over time. In TD girls, there were no differences at baseline in ED features and psychopathology between the pre- and peri-pandemic group.

**Conclusion:**

Overall, peri-pandemic AN participants were less severely ill, compared to pre-pandemic AN participants, which may be explained by less social pressure and peer contact, and a more protective parenting style during the pandemic. Conversely, peri-pandemic AN participants had a less favorable clinical course, which may be explained by reduced access to health care facilities during the pandemic.

**Level of evidence:**

Level III: Evidence obtained from well-designed cohort or case–control analytic studies.

**Supplementary Information:**

The online version contains supplementary material available at 10.1007/s40519-024-01640-0.

## Introduction

Anorexia nervosa (AN) is a life-threatening mental disorder characterized by an extremely low body weight, a distorted body image and an intense fear to gain weight [1]. The onset of AN is often during adolescence and its lifetime prevalence is around 1–4% in women and 0.2–0.3% in men [2, 3]. AN is challenging to treat and the often long road to recovery focuses on weight restoration and psychotherapy [4]. Several risk factors have been identified, including psychosocial factors such as stressful life events which not only influence the susceptibility to develop an eating disorder (ED), but also have implications for the course of the disease, i.e., severity and duration [5]. A recent example of a traumatic stressor is the COVID-19 pandemic, which had a major impact on the mental health of the global community [6, 7]. To limit the transmission of the virus, governments implemented extensive measures, including social distancing, closure of non-essential services, and limitations in social interactions [8]. The mental health of adolescents was a main concern during the pandemic [9, 10], since adolescence is a formative period for socio-emotional, cognitive and coping skills development [11]. Therefore, adolescents with AN especially may have been extra vulnerable and susceptible to the harmful effects of the pandemic [12].

Since the onset of the COVID-19 pandemic, an increase of 15% in ED presentations [13], 83% in pediatric ED hospital admissions and 16% in adult admissions [14] has been reported. Despite the relatively larger increase in pediatric admissions, previous studies have mainly focused on adults who had been diagnosed with AN prior to the pandemic. Overall, these studies showed mixed results [12, 14, 15]. Some studies found a worsening of ED symptoms [16–19], and increased levels of depression- and anxiety symptoms [17, 20, 21], while others showed an improvement in ED symptoms, more social support, and more time for self-reflection in the pandemic period compared to pre-pandemic [22–26]. Springall et al. [27] conducted in adolescents, a large retrospective chart review from 2017 to 2020 of all individuals with AN presenting to a children’s hospital, with respect to hospitalization outcomes, body mass index (BMI), and comorbid psychopathology. Despite the increase in ED presentations peri-pandemic, they found no year-over-year differences in disorder severity. Other studies comparing pre-pandemic and peri-pandemic adolescent AN cohorts have shown conflicting results. One cross-sectional prospective study found no differences in ED symptomatology, but did find lower BMI and more compensatory over-exercising in the peri-pandemic group [28]. Another retrospective chart-review study, found no differences in BMI, but found greater medical instability and higher hospitalization rates in the peri-pandemic AN cohort compared to the pre-pandemic AN cohort [29]. Meneguzzo et al. [30] discovered that the peri-pandemic AN cohort had an increase in binge eating episodes, more difficulties with interoceptive awareness, and fewer obsessive–compulsive disorder (OCD) symptoms compared to the pre-pandemic AN cohort.

Our study addresses several knowledge gaps that may help to improve our understanding of the impact of the COVID-19 pandemic on illness severity and disease course among adolescents with first-onset clinical AN. First, previous work of Springall [27], Spettigue [29], and Meneguzzo [30] was based on a retrospective chart review in which data were not systematically collected, whereas our study extensively assessed AN participants at two timepoints (at baseline and after 1 year of follow-up) in multiple domains during face-to-face appointments using structured interviews. Second, we are the first study to examine the one-year clinical course of adolescent AN during the pandemic. Finally, we used an age-, gender- and education-matched typically developing (TD) group as a reference cohort. Our findings may be helpful in the clinical management of adolescents with AN during stressful situations such as the COVID-19 pandemic.

In this context, we aim to investigate the implications of the COVID-19 pandemic in adolescents with AN. The current study is embedded in the BRAVE study; the BRAVE study is a first-onset AN prospective longitudinal cohort study focusing on the following four research areas: (1) behavior, (2) neurobiology, (3) cognitive functions, and (4) physical health. As the onset of the COVID-19 pandemic occurred in the middle of data collection, our study provided a natural experiment in which we could examine the impact of the COVID-19 pandemic on ED features and comorbid psychopathology in a large group of adolescent females with first-onset AN.

We hypothesized that AN participants enrolled peri-pandemic would have more severe ED, i.e., a lower BMI and more ED symptoms, and experience more psychopathology compared to AN participants enrolled pre-pandemic. We expected that TD girls enrolled peri-pandemic would experience more ED symptoms and psychopathology, albeit at a subclinical level, compared to TD girls enrolled pre-pandemic. Second, to examine the effect of the COVID-19 pandemic on the one-year disease course, we examined the longitudinal association between ED features and the timing of the pandemic during the one-year follow-up period, i.e., both the pre-pandemic and peri-pandemic time-periods.

## Materials and methods

### Participants

The sample consisted of a total of 154 female adolescents: 79 participants with first-onset AN and 75 TD girls. AN inclusion criteria included: female gender, aged 12–22 years, diagnosed with first-onset AN or atypical AN according to DSM-5 criteria. The onset of the DSM-5 diagnosis of AN should be within 12 months of the date of inclusion. AN participants were recruited from 16 mental health institutions and hospitals in the Netherlands via the providers. In addition, they were recruited via advertisements on social media and through patient organizations.

An age-, gender-, and education-matched TD group was also included. To be included, TD girls were required to have a healthy body weight, defined as a body mass index—standard deviation score (BMI-SDS) between − 1.3 and + 1.3. TD girls were recruited via advertisements on social media, sport clubs or via AN participants (e.g., classmates, friends). Exclusion criteria for both the AN and TD group were presence of a psychotic-, neurologic-, or a substance abuse disorder, severe motor and sensory disturbances, IQ < 70 measured by an intelligence test, and insufficient Dutch language skills.

### Study design

The study was conducted between May 2017 and January 2023. The study had a longitudinal design with identical measurements performed at baseline (T1) and after 1 year of follow-up (T2). As previous studies have shown that the COVID-19 restrictions in particular have had a major impact on people’s lives [31–33], the cut-off point (pre- versus peri-pandemic) in this study was set at the 15th of March, 2020, the date on which the Dutch government announced the first major restrictions.

**Fig. 1:**
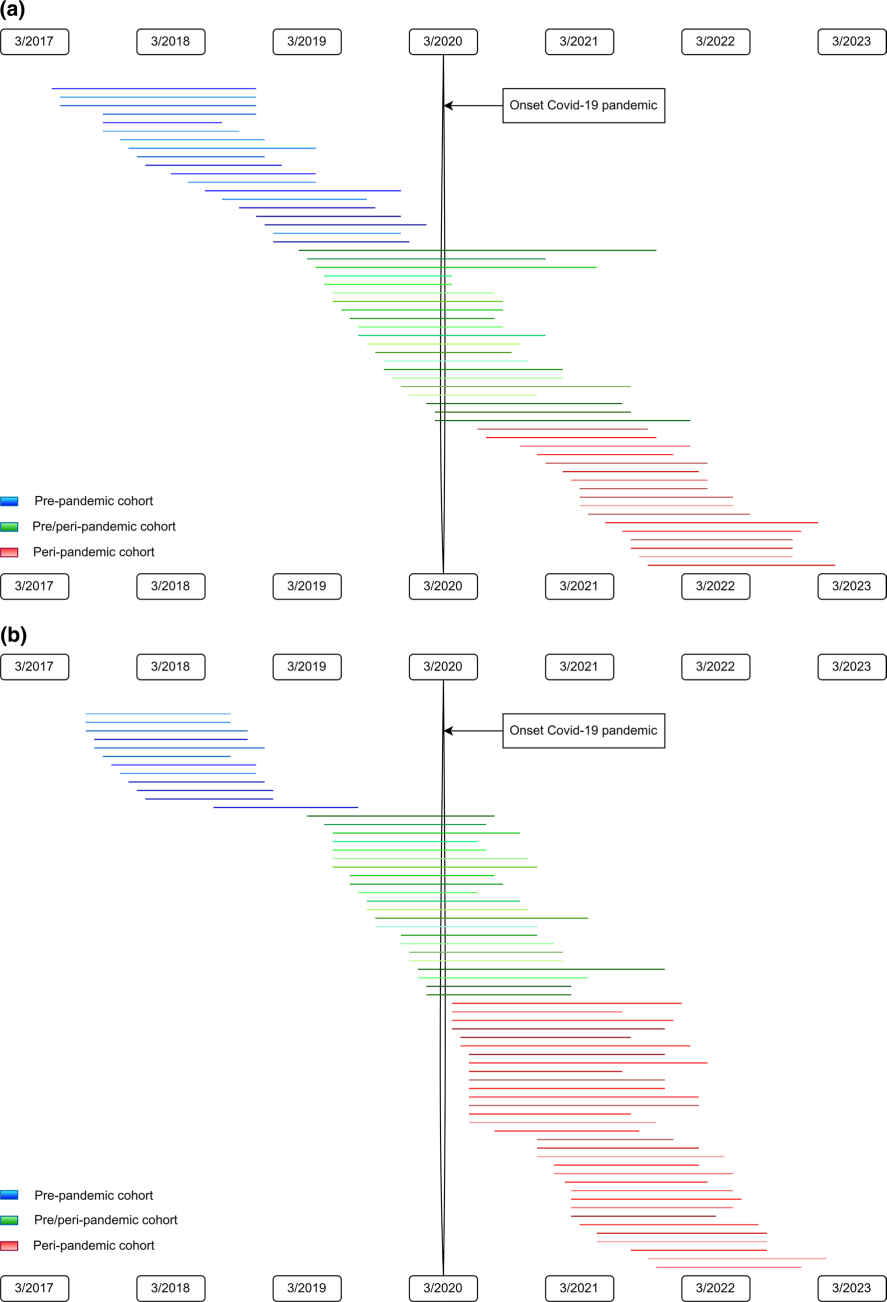
**a** Overview of pre-pandemic, pre/peri-pandemic, and peri-pandemic AN participants. AN: anorexia nervosa. Each line represents the trajectory of each individual AN participant in the BRAVE study. **b** Overview of pre-pandemic, pre/peri-pandemic, and peri-pandemic TD girls. TD: typically developing. Each line represents the trajectory of each individual TD participant in the BRAVE study

ED features and psychopathology at T1 were compared between separate groups: a pre-pandemic cohort (assessed between 3/5/2017 and 15/3/2020) and a peri-pandemic cohort (assessed between 16/3/2020 and 14/10/2021). The AN group consisted of 49 participants enrolled pre-pandemically and 30 participants enrolled peri-pandemically. The TD group consisted of, respectively, 38 participants enrolled pre- and 37 enrolled peri-pandemically. T2 data were available for 72% (*n* = 57) of AN participants and 88% (*n* = 66) of TD girls. To explore the longitudinal association between timing of the pandemic and ED features, we compared three groups: (1) Pre-pandemic cohort (AN, *n* = 19; TD, *n* = 12), in which all data were collected pre-pandemically; (2) pre/peri-pandemic cohort (AN, *n* = 21, TD, *n* = 22), in which inclusion was pre-pandemic and 1-year follow-up occurred peri-pandemically; (3) peri-pandemic cohort (AN, *n* = 17; TD, *n* = 32), in which all data were collected peri-pandemically (Fig. 1a and b).

### Procedure

The eligibility of adolescents who were interested in participating in our study was assessed by screening inclusion and exclusion criteria by telephone. After the screening, potential participants were provided information about the study and were contacted again 2 weeks later to answer any remaining questions. If the adolescent wished to participate, a consent meeting was scheduled to obtain informed consent and verify eligibility. For both the TD and AN groups, we assessed inclusion and exclusion criteria by using the Mini International Neuropsychiatric Interview (MINI)-KID/PLUS to check for psychotic disorders and substance-related disorders. For the AN participants, DSM-5 criteria for AN were evaluated, and interviews/questionnaires were administered focusing on the following research domains: (1) behavior, (2) neurobiology, (3) cognitive functions, and (4) physical health. Two visits at the Erasmus MC-Sophia Children’s Hospital were planned in random order. During one visit neuropsychological assessments, including intelligence tests and cognitive tasks, were administered. During the second visit, mental health measures were obtained. Participants also completed online questionnaires that included ED-related questionnaires, sociodemographic information, and psychopathology related questionnaires. Between T1 and T2 AN participants received treatment as usual. To determine eligibility for TD girls, we assessed their height and body weight to ensure they met the criteria for a healthy weight. Additionally, we screened them for the presence of eating disorders. Our aim was to include a control group that accurately represents the general population in terms of the prevalence of psychopathology. TD girls underwent the same procedure as AN participants.

### Materials

#### Primary outcomes

*BMI-SDS.* BMI was corrected for age and gender to obtain an adjusted BMI (BMI-SDS), using a growth calculator tool (https://groeiweb.pgdata.nl/calculator.asp).

*ED symptomatology.* The Eating Disorder Examination version 12.0 (EDE) is considered the gold standard for assessing ED psychopathology [34], consisting of four subscales (rated on a 7-point Likert scale): dietary concerns, food concerns, body weight concerns and body shape concerns. The internal consistency of the global score was high (α = 0.92) [35].

In addition, ED symptoms were assessed using the Eating Disorder Inventory, version 3 (EDI-3) [36], a self-report questionnaire consisting of 91 items, each rated on a 4-point Likert scale spanning 12 subscales. Higher scores indicate an increased risk of having or developing an ED. The EDI questionnaire demonstrates high internal consistency, with a range above α = 0.80 [37].

*Body shape concerns*. The Body Shape Questionnaire (BSQ) is a self-report questionnaire that was used to assess body shape concerns typical for ED symptoms in the prior 4 weeks [38]. The BSQ consists of 34 items scored on a 6-point Likert scale. Higher scores indicate greater perceived body shape dissatisfaction. The internal consistency among adolescents is high (α = 0.97) [39].

*Physical exercise.* Time spent on physical exercise during a typical week was recorded, divided into high and low intensity. High-intensity physical exercise was defined as vigorous activity (e.g., running, swimming). Low-intensity physical exercise was low impact (e.g., walking, yoga).

*Hand grip strength.* Muscle strength was measured using a hand grip instrument (JAMAR®-plus + Digital Hand Dynamometer) set to kg; averaged over three trials.

*Arm circumference,* averaged over both upper arms.

#### Secondary outcomes

*General psychopathology.* The Mini International Neuropsychiatric Interview (MINI-KID (< 17 years) /MINI-PLUS (> 18 years) is a structured interview used to assess general psychopathology based on DSM-IV criteria. The MINI-KID/MINI-PLUS based on DSM-5 criteria was not available at the start of our study. Test–retest kappa (*k* = 0.76–0.93) and the specificity for all diagnoses (range 0.72–0.92) is moderate–high [40].

*Depression.* The Beck Depression Inventory-Second Edition (BDI-II) was used to measure severity of depressive symptoms using statements reflecting mood symptoms within the past week [41], consisting of 21 items, rated on a 4-point Likert scale. A score of 0–20 points indicates ‘no or minimal depression’, a score of 21–30 ‘moderate depression’, a score of 31–40 ‘severe depression’ and a score of 41–63 ‘very severe depression’. The internal consistency is high (α = 0.9) and the test–retest reliability is moderate–high (range 0.73–0.96) [42].

*Anxiety.* The Screening for Child Anxiety Related Emotional Disorders (SCARED) assessed anxiety symptoms using DSM-IV-TR criteria [43]. The SCARED consists of 69 items scored on a 3-point Likert scale. Scores above 25 were considered indicative of an anxiety disorder [44]. Internal consistency is moderate–high (α = 0.74–0.93) [45].

*Obsessive–compulsive disorder (OCD).* The (Children’s Yale-Brown Obsessive–Compulsive Scales ((C)Y-BOCS) questionnaire assesses obsessive–compulsive thoughts and behaviors [46]. The CY-BOCS was administered to ages 12–17 year olds and the Y-BOCS to 18–22 year olds. This questionnaire consists of 10 items, scored on a 5-point Likert scale. The Y-BOCS has a high internal consistency (α = 0.87) [47].

*Autism.* The Social Responsiveness Scale (SRS) assesses autistic traits [48]. The SRS-2 was completed by parents in participants aged 12–17 years; the SRS-A was used in participants aged 18–22 years. The internal consistency of the SRS-2 is high (α = 0.91) and the interrater reliability ranging between α = 0.76 and α = 0.95 [48]. The internal consistency of the SRS-A is also high (α = 0.89) in adults with autism spectrum disorder (ASD) and moderate (α = 0.71) in TD adults [49].

*Intelligence.* Intelligence was assessed using the Wechsler Abbreviated Scale of Intelligence-Second Edition (WASI-II) [50]. The resulting Full Scale Intelligence Quotient (FSIQ) provides a reliable estimate of cognitive functioning [51]. The internal consistency is high (α = 0.87–0.91) [50].

### Covariates

Age and socioeconomic status (SES) were considered as covariates. The SES was based on the education of the mother. Maternal education was divided into: low (primary education and lower general secondary education), middle (higher general secondary education), and high (higher vocational secondary education or University degree).

### Statistical analyses

Data analyses were performed using SPSS Statistics (version 28.0, IBM Corporation, Armonk, NY, USA). Effect sizes were reported as partial eta squared (η^2^_p_), with 0.01 indicating a small effect size, 0.06 a medium effect size and 0.14 a large effect size [52].

#### Cross-sectional analyses

Sociodemographic variables were analyzed using a 2-sided independent Student’s t-test or a Mann–Whitney U test for non-parametric data. Separate analyses were performed for the AN and TD groups. A Kruskal–Wallis one-way ANOVA was used to compare group differences in ethnicity, SES and recruitment method.

For comparisons between AN participants, pre-pandemic and peri-pandemic measures of emotional dysregulation (EDI subscale) and physical exercise were log-transformed to approximate a normal distribution. For the analyses within the TD group, measures of ED symptomatology (EDE and EDI scores), body shape satisfaction (BSQ), obsessive–compulsive symptoms (Y-BOCS), and physical exercise were log-transformed to approximate a normal distribution. Comparisons between ED features and psychopathology symptoms were made using one-way analyses of covariance (ANCOVA), corrected for age and SES. The independent variable was group (pre- vs. peri-pandemic) and the dependent variables were ED features and comorbid psychiatric symptoms. Comparisons between number of DSM-IV diagnoses were analyzed using Pearson Chi-square tests.

In addition, we performed a double subtraction analysis in order to perform a case–control comparison of pre- versus peri-pandemic. For both pre- and peri-pandemic analyses measures of ED—(EDE), OCD—(Y-BOCS), ASD symptoms (SRS-2/SRS-A), and physical exercise were log-transformed in order to approximate a normal distribution. We performed multiple ANCOVAs to test for differences between ED features and comorbid psychopathology between AN and TD participants both pre- and peri-pandemic. Group differences in the number of DSM-IV diagnoses were carried out using Pearson Chi-squared tests. Correction for multiple testing was performed by controlling for false discovery rate (FDR) at a q-value of 0.05.

#### Longitudinal analyses

To examine the longitudinal association between ED features and pandemic timing (pre-pandemic vs. peri-pandemic), we calculated a mean difference score between T1 and T2 for the pre-pandemic, pre/peri-pandemic and peri-pandemic cohorts. Longitudinal comparisons for the main clinical outcomes (BMI-SDS and ED symptomatology), were performed using ANCOVAs adjusted for age, SES, follow-up time, and baseline BMI-SDS and EDE scores, respectively. Post hoc analyses were performed to assess differences in the course of ED features between the pre-pandemic, pre/peri-pandemic, and peri-pandemic subgroups.

#### Non-response analyses

AN and TD participants with missing data at follow-up were compared with participants with available follow-up data on age, SES, ED symptoms and BMI-SDS.

## Results

### Sample characteristics

Sociodemographic characteristics are shown in Table 1. The SES of peri-pandemic AN participants was higher compared to pre-pandemic AN participants (*p* < 0.05). There were no differences in age, ethnicity, IQ, and duration of illness between pre- and peri-pandemic AN participants. All pre-pandemic AN participants (100%) were recruited via health care providers, whereas 60% of the peri-pandemic AN participants were recruited through their health care providers (*n* = 18). The remainder were recruited via social media 33.3% (*n* = 10) or via flyers and other means 2.7% (*n* = 2). Of note, ED features and comorbid psychopathology did not differ between peri-pandemic recruitment methods (Supplemental Table 1). There were no differences in age, ethnicity, SES, and IQ between TD girls enrolled pre- and peri-pandemic. In the non-response analyses, AN participants with missing follow-up data (*n* = 22) were compared with AN participants with available follow-up data (*n* = 57). AN participants with missing data at follow-up had less severe ED symptoms at baseline compared to AN participants for whom data were available (*p* = 0.04). Pre- and peri-pandemic AN participants did not differ in age and SES. TD participants with missing data at follow-up (*n* = 8) were compared with TD participants for whom follow-up data were available (*n* = 67). There was no selective drop-out based on age, SES, ED symptoms and BMI-SDS.

**Table 1:** Sociodemographic characteristics of participants of the BRAVE study pre- and peri-pandemic

			AN participants (*n* = 79)				TD participants (*n* = 75)		
	*N*	Statistic	Pre-pandemic (*n* = 49)	Peri-pandemic (*n* = 30)	*p* value	*N*	Pre-pandemic (*n* = 37)	Peri-pandemic (*n* = 38)	*p* value
Age (years)	79	Mean (SD)	16.08 (2.06)	16.99 (2.41)	0.152	75	17.29 (2.34)	17.19 (2.16)	0.299
Ethnicity	75	Percentage			0.305	74			0.985
Dutch			97.8	93.1			97.2	97.4	
Western			2.2	3.4			2.8	0	
Non-Western			0	3.4			0	2.6	
SES^A^	73	Percentage			0.047*	70			0.344
Low			15.9	10.3			2.9	8.6	
Middle			40.9	20.7			42.9	22.9	
High			43.2	69.0			54.3	68.6	
FSIQ total score	78	Mean (SD)	106.67 (12.67)	113.34 (10.71)	0.435	75	112.41 (12.72)	110.82 (10.67)	0.176
Duration of illness (months)	71	Mean (SD)	4.72 (3.77)	5.48 (3.03)	0.116	–	–	–	–

^A^SES: socioeconomic status based on maternal education; categorized into: Low: primary education and lower general secondary education; Middle: higher general secondary education; High: higher vocational secondary education and higher academic education; AN: anorexia nervosa; FSIQ-4: Full Scale Intelligence Quotient. TD: typically developing. *p < 0.05

### Cross-sectional baseline comparisons of ED features and comorbid psychopathology pre-pandemic and peri-pandemic

#### Case–case comparisons

**Fig. 2:**
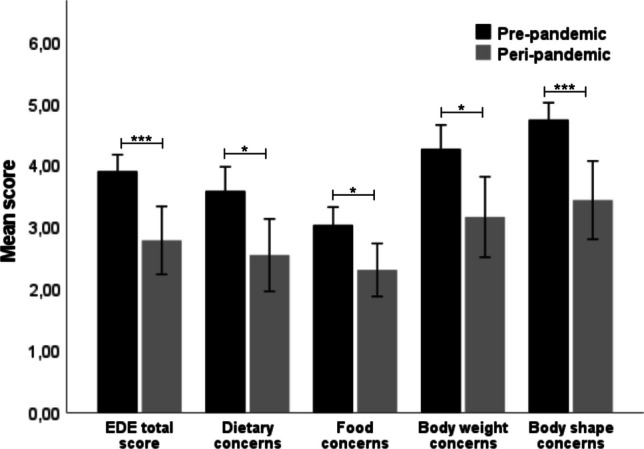
ED symptoms (total score and subscale scores) of AN participants assessed by the Eating Disorder Examination pre-pandemic and peri-pandemic*.* EDE: Eating Disorder Examination. *p < 0.05, **p < 0.01, ***p < 0.001

AN participants recruited peri-pandemic reported fewer ED symptoms at enrollment, as assessed by the EDE compared to AN participants recruited pre-pandemic (*p* =  < 0.001). Further EDE analyses revealed that peri-pandemic AN participants experienced fewer dietary concerns (*p* = 0.007), food concerns (*p* = 0.005), body weight concerns (*p* = 0.009), and body shape concerns (*p* =  < 0.001) compared to pre-pandemic AN participants (Tables 2 and 3, Fig. 2). The effect sizes of these findings were moderate to large (η^2^_p_ 0.10–0.17). Peri-pandemic AN participants experienced less interpersonal insecurity (EDI-3 subscale Interpersonal insecurity*: p* = 0.001), and reported being less dissatisfied with their body shape (EDI-3 subscale Body dissatisfaction: *p* = 0.007) in comparison with pre-pandemic AN participants. The effect sizes were moderate to large (η^2^_p_ 0.11–0.15). AN participants included peri-pandemic had less hand grip strength (*p* = 0.034) and a lower arm circumference (*p* = 0.032) than AN participants included pre-pandemic, although this finding was no longer significant after multiple-testing correction. There was no difference in BMI-SDS between AN participants included pre- and peri-pandemic. AN participants recruited peri-pandemic fulfilled less DSM-IV classifications compared to AN participants recruited pre-pandemic (*(X*^*2*^*)* = 13.29, *df* = 5, *p* < 0.021) (Supplemental Fig. 1), primarily driven by less classifications for anxiety disorders ((*X*^*2*^*)* = 21.72, *df* = 1, *p* < 0.001; Supplemental Table 3). There were no differences in depression-, anxiety-, OCD- and ASD symptoms between AN participants included pre- and peri-pandemic.

**Table 2:** Eating disorder features of TD and AN participants pre- and peri-pandemic

	AN participants (*n* = 79)						TD participants (*n* = 75)					
	*N*	Pre-pandemic (*n* = 49)	Peri-pandemic (*n* = 30)	ANCOVA’s	*p* value	η^2^_p_	*N*	Pre-pandemic (*n* = 37)	Peri-pandemic (*n* = 38)	ANCOVA’s	*p* value	η^2^_p_
		Mean (SD)	Mean (SD)					Mean (SD)	Mean (SD)			
BMI-SDS	73	− 1.29 (1.21)	− 1.25 (1.40)	*F*(1.67) = 0.67	0.402	0.01	72	0.28 (0.83)	0.61 (1.01)	*F*(1.64) = 2.24	0.140	0.03
Hand grip strength	60	24.49 (4.66)	22.11 (4.87)	*F*(1.55) = 4.72	0.032	0.08	61	25.05 (6.40)	24.67 (4.45)	*F*(1.52) = 0.10	0.753	0.00
Arm circumference	60	231.80 (27.73)	213.35 (25.16)	*F*(1.55) = 4.84	0.034	0.08	60	248.26 (15.96)	252.43 (28.57)	*F*(1.51) = 0.24	0.630	0.01
Physical exercise^‡^												
Low intensity	53	3.27 (3.98)	5.40 (6.41)	*F*(1.41) = 0.26	0.615	0.01	61	2.79 (2.46)	3.36 (2.26)	*F*(1.48) = 1.92	0.172	0.04
High intensity	53	1.92 (2.34)	1.96 (2.14)	*F*(1.48) = 0.25	0.624	0.05	61	4.09 (2.83)	3.08 (3.32)	*F*(1.45) = 0.15	0.146	0.05
EDE total score	79	3.90 (0.93)	2.78 (1.47)	*F*(1.69) = 13.88	< 0.001*	0.17	75	0.33 (0.73)	0.47 (0.48)	*F*(1.55) = 1.80	0.185	0.03
EDI total score	68	157.28 (40.64)	146.50 (42.76)	*F*(1.62) = 0.95	0.333	0.02	71	41.50 (22.16)	45.73 (32.03)	*F*(1.64) = 0.18	0.676	0.00
BSQ total score	69	143.46 (31.17)	122.43 (34.11)	*F*(1.63) = 5.25	0.025	0.08	70	50.67 (16.98)	57.51 (18.42)	*F*(1.63) = 2.15	0.147	0.03

AN: anorexia nervosa; TD: typically developing; BMI-SDS: body mass index—standard deviation score; EDE: Eating Disorder Examination; EDI: Eating Disorder Inventory; BSQ: Body Shape Questionnaire. *significant *p*-value after correction for multiple testing using FDR with a cut-off point of 0.05. ^‡^Data available from a limited number of participants, because these measures were no part of the study protocol until 24/10/2017

**Table 3:** Eating disorder symptomatology—subscales of TD and AN participants pre- and peri-pandemic

		AN participants (*n* = 79)						TD participants (*n* = 75)				
	*N*	Pre-pandemic (*n* = 49)	Peri-pandemic (*n* = 30)	ANCOVA’s	*p* value	η^2^_p_	*N*	Pre-pandemic (*n* = 37)	Peri-pandemic (*n* = 38)	ANCOVA’s	*p* value	η^2^_p_
		Mean (SD)	Mean (SD)					Mean (SD)	Mean (SD)			
EDE total score	79	3.90 (0.93)	2.78 (1.47)	*F*(1.69) = 13.88	< 0.001*	0.17	75	0.33 (0.73)	0.47 (0.48)	*F*(1.55) = 1.80	0.185	0.03
Dietary concerns	79	3.58 (1.36)	2.55 (1.57)	*F*(1.69) = 7.72	0.007*	0.10	75	0.18 (0.46)	0.40 (0.52)	*F*(1.23) = 0.24	0.626	0.01
Food concerns	79	3.03 (1.01)	2.31 (1.15)	*F*(1.69) = 8.23	0.005*	0.11	75	0.06 (0.24)	0.13 (0.34)	*F*(1.14) = 0.45	0.515	0.03
Body weight concerns	79	4.27 (1.35)	3.16 1.74)	*F*(1.69) = 7.33	0.009*	0.10	75	0.27 (0.58)	0.57 (0.70)	*F*(1.33) = 1.63	0.211	0.05
Body shape concerns	79	4.74 (0.95)	3.44 (1.70)	*F*(1.69) = 13.99	< 0.001*	0.17	75	0.36 (0.52)	0.55 (0.67)	*F*(1.44) = 0.48	0.494	0.01
EDI total score	68	157.28 (40.64)	146.50 (42.76)	*F*(1.62) = 0.95	0.333	0.02	71	41.50 (22.16)	45.73 (32.03)	*F*(1.64) = 0.18	0.676	0.00
Drive for thinness	68	22.08 (6.51)	20.04 (7.90)	*F*(1.62) = 1.29	0.260	0.02	71	1.59 (2.90)	2.41 (3.34)	*F*(1.28) = 0.10	0.751	0.00
Bulimia	68	2.98 (4.89)	2.36 (3.78)	*F*(1.34) = 0.38	0.540	0.01	71	0.41 (0.74)	2.49 (3.84)	*F*(1.25) = 3.35	0.079	0.12
Body dissatisfaction	68	31.20 (7.07)	25.25 (7.66)	*F*(1.62) = 7.85	0.007*	0.11	71	5.79 (6.10)	7.43 (6.46)	*F*(1.50) = 0.35	0.558	0.01
Low self-esteem	68	14.13 (4.66)	11.57 (5.58)	*F*(1.62) = 3.22	0.078	0.05	71	2.47 (2.65)	2.08 (2.20)	*F*(1.45) = 0.78	0.382	0.02
Personal Alienation	68	12.80 (4.54)	12.46 (5.09)	*F*(1.62) = 0.02	0.896	0.00	71	3.08 (2.64)	3.35 (3.74)	*F*(1.47) = 0.40	0.529	0.01
Interpersonal Insecurity	68	12.23 (4.75)	8.04 (5.25)	*F*(1.62) = 11.21	0.001*	0.15	71	4.76 (4.45)	4.08 (3.49)	*F*(1.50) = 0.09	0.767	0.00
Interpersonal Alienation	68	9.25 (4.60)	8.04 (5.20)	*F*(1.62) = 0.82	0.370	0.01	71	3.50 (3.21)	3.76 (3.90)	*F*(1.50) = 0.06	0.814	0.00
Interoceptive Deficits	68	13.90 (6.32)	16.00 (7.62)	*F*(1.62) = 0.80	0.374	0.01	71	2.82 (3.00)	3.46 (4.71)	*F*(1.45) = 0.06	0.814	0.00
Emotional Dysregulation	68	5.03 (5.17)	5.61 (4.74)	*F*(1.58) = 1.95	0.168	0.03	71	1.59 (1.86)	2.05 (2.97)	*F*(1.38) = 0.51	0.478	0.01
Perfectionism	68	9.53 (4.60)	11.93 (5.80)	*F*(1.62) = 2.06	0.156	0.03	71	4.23 (3.29)	5.32 (4.16)	*F*(1.61) = 0.29	0.593	0.01
Asceticism	68	12.35 (6.28)	12.00 (6.29)	*F*(1.62) = 0.05	0.833	0.00	71	1.62 (1.60)	2.46 (3.67)	*F*(1.43) = 0.00	0.976	0.00
Maturity fears	68	11.83 (6.13	13.21 (6.77)	*F*(1.62) = 0.57	0.452	0.01	71	9.62 (4.53)	6.84 (4.14)	*F*(1.62) = 5.6	0.021	0.08

AN: anorexia nervosa; TD: typically developing; EDE: Eating Disorder Examination; EDI: Eating Disorder Inventory; *: significant *p*-value after correction for multiple testing using FDR with a cut-off point of 0.05

#### Control–control comparisons

Peri-pandemic TD girls perceived less maturity fears (EDI-3 scale Maturity fears: *p* = 0.004) than pre-pandemic TD girls, although no longer significant after multiple-testing correction. There were no other differences in ED features and in depression-, anxiety-, OCD- and ASD symptoms between TD girls included pre- and peri-pandemic (Table 4).

**Table 4:** Comorbid psychiatric psychopathology of AN and TD participants pre- and peri-pandemic

			AN participants (*n* = 79)						TD participants (*n* = 75)				
	*N*	Statistic	Pre-pandemic (*n* = 49)	Peri-pandemic (*n* = 30)	ANCOVA’s	*p* value	η^2^_p_	*N*	Pre-pandemic (*n* = 37)	Peri-pandemic (*n* = 38)	ANCOVA’s	*p* value	η^2^_p_
Depression (BDI-II)	65	Mean (SD)	27.00 (9.39	22.79 (11.82)	*F*(1.60) = 3.11	0.083	0.05	71	4.53 (4.45)	4.59 (5.06)	*F*(1.64) = .11	0.740	0.00
Anxiety (SCARED)	65	Mean (SD)	45.91 (21.07)	41.54 (22.29)	*F*(1.60) = 0.34	0.561	0.01	71	21.41 (14.13)	23.03 (15.76)	*F*(1.64) = .01	0.907	0.00
Compulsory thoughts (Y-BOCS)	63	Mean (SD)	3.51 (5.99)	4.82 (5.63)	*F*(1.56) = 2.38	0.129	0.04	67	0.03 (0.18)	0.84 (2.92)	*F*(1.60) = 1.37	0.247	0.02
Compulsory habits (Y-BOCS)	63	Mean (SD)	5.14 (6.67)	4.68 (5.52)	*F*(1.56) = 0.03	0.863	0.00	67	0.63 (2.01)	0.51 (2.21)	*F*(1.60) = 1.03	0.314	0.02
Autism (SRS-2)	54	Mean (SD)	56.71 (10.22)	51.89 (7.59)	*F*(1.48) = 2.27	0.139	0.05	48	44.14 (6.33)	48.19 (11.14)	*F*(1.42) = 1.14	0.292	0.03
Autism (SRS-A)	11	Mean (SD)	72.00 (1.73)	71.50 (5.32)	*F*(1.8) = 0.03	0.874	0.00	17	68.09 (1.45)	68.17 (1.17)	*F*(1.13) = .08	0.779	0.01
DSM-IV classifications (MINI-interviews)													
Any mood disorder	49	Percentage	69.4	51.7	–	0.119		10	13.9	13.2	–	0.927	
Any anxiety disorder	35	Percentage	61.2	17.2	–	< 0.001*		9	11.1	10.5	–	0.935	
Any OCD	17	Percentage	20.4	24.1	–	0.700		1	0	2.6	–	0.327	
Any behavior disorder	3	Percentage	2.0	6.9	–	0.281		0	0	0	–	–	
Any ADHD	4	Percentage	4.1	6.9	–	0.586		3	2.8	5.3	–	0.588	
Any ASD	0	Percentage	0	0	–	–		0	0	0	–	–	

AN: anorexia nervosa; TD: typically developing; BDI-II: Beck Depression Inventory-Second edition; SCARED: Screening for Child Anxiety Related Emotional Disorders; Y-BOCS: Yale-Brown Obsessive–Compulsive Scales; SRS-2: Social Responsiveness Scale-Second edition. SRS-A: Social Responsiveness Scale-Adults. OCD: obsessive compulsive disorder; ADHD: attention deficit hyperactivity disorder; ASD: autism spectrum disorder. *: significant *p*-value after correction for multiple testing using FDR with a cut-off point of 0.05

#### Case–control comparisons

AN participants had a lower BMI-SDS, arm-circumference, EDE-, EDI-, and BSQ-score compared to TD girls, both pre- and peri-pandemic (*p* < 0.05, Supplemental Table S).

Pre- and peri-pandemic, AN participants had higher depression-, anxiety-, and ASD symptoms compared to TD girls (*p* < 0.05). Pre-pandemic, AN participants displayed significantly more OCD symptoms than TD girls, similar to peri-pandemic although this difference was no longer significant due to a slight increase in OCD symptoms in TD girls peri-pandemic.

### Longitudinal association between ED features and timing of the pandemic during the one-year follow-up period

#### Case–case comparisons

**Fig. 3:**
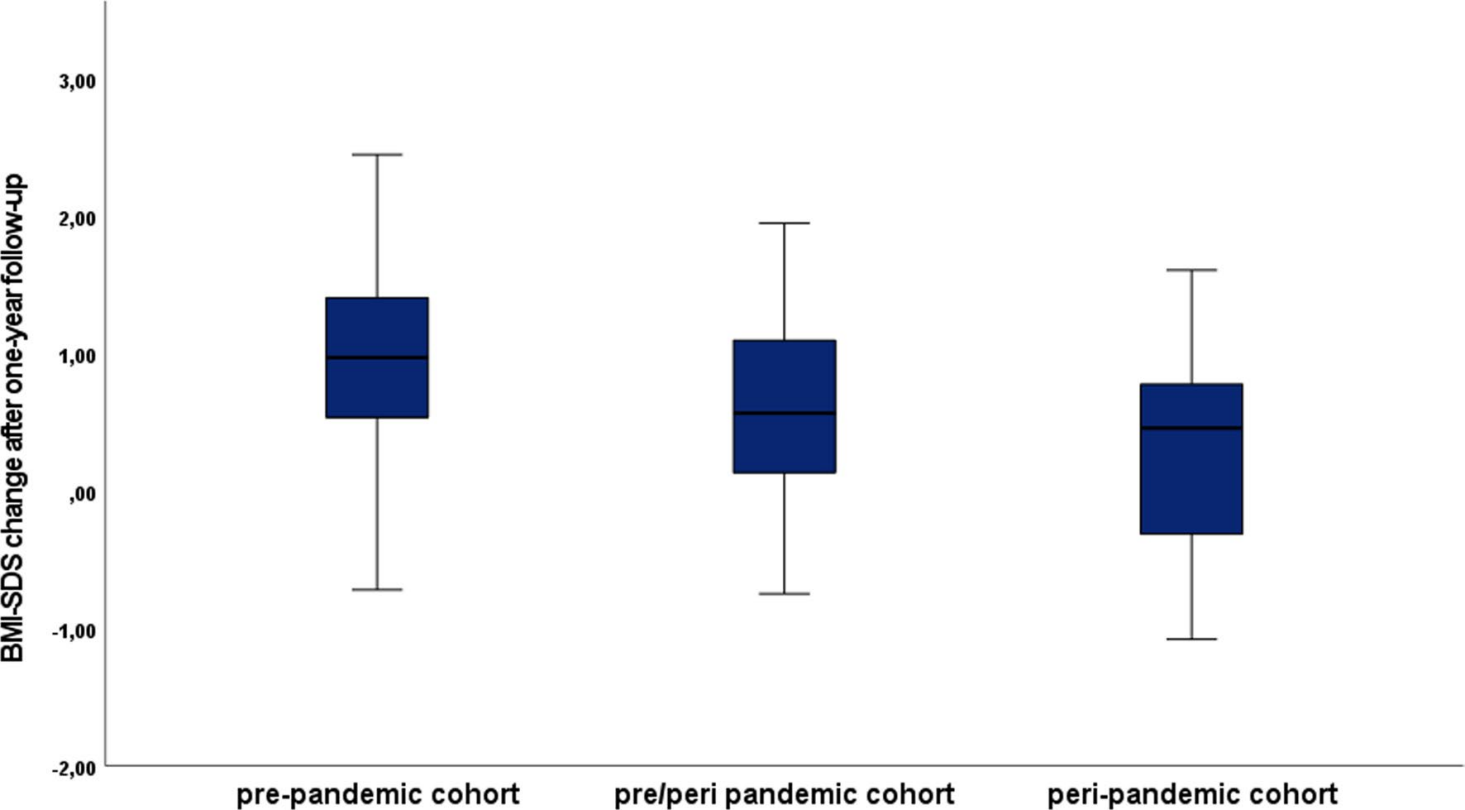
BMI-SDS change over the course of 1 year of follow-up among AN participants

Within the AN group, the peri-pandemic group had a lower BMI-SDS increase between baseline and follow-up compared to the pre-pandemic AN group and the pre/peri-pandemic AN group (*p* = 0.028) (Fig. 3, Table 5). There were no significant between-group differences in ED symptoms over time.

**Table 5:** ED features and psychopathology over time

		Pre-pandemic cohort				Pre/peri-pandemic cohort				Peri-pandemic cohort					
	*N*	T1	T2	Change over time	*N*	T1	T2	Change over time	*N*	T1	T2	Change over time	ANCOVA’s	*p* value	η^2^_p_
		Mean (SD)	Mean (SD)	Mean (SD)		Mean (SD)	Mean (SD)	Mean (SD)		Mean (SD)	Mean (SD)	Mean (SD)			
AN participants															
BMI-SDS	18	− 1.76 (0.87)	− 0.89 (1.05)	0.85 (0.85)	21	− 0.87 (1.34)	− 0.27 (1.31)	0.59 (0.73)	16	− 1.39 (1.13)	− 1.16 (0.92)	0.26 (0.76)	*F*(2.47) = 3.87	0.028*	0.14
EDE total score	19	3.87 (0.89)	2.15 (1.47)	− 1.71 (1.46)	20	3.77 (1.03)	2.67 (1.29)	− 1.17 (1.42)	17	2.99 (1.44)	2.29 (1.22)	− 0.70 (1.05)	*F*(2.46) = 2.33	0.675	0.02
TD participants															
BMI-SDS	12	0.45 (0.92)	0.50 (0.92)	0.05 (0.25)	22	0.15 (0.80)	0.25 (1.04)	0.10 (0.73)	31	0.59 (0.92)	0.64 (0.99)	− 0.06 (.45)	*F*(2.54) = 0.18	0.837	0.01
EDE total score	12	0.30 (0.51)	0.28 (0.45)	− 0.01 (0.21)	21	0.36 (0.87)	0.30 (0.49)	− 0.06 (0.91)	32	0.44 (0.49)	0.43 (0.55)	− 0.01 (.46)	*F*(2.55) = 1.61	0.879	0.01

AN: anorexia nervosa; TD: typically developing; BMI-SDS: body mass index—standard deviation score; EDE: Eating Disorder Examination; *significant *p*-value with a cut-off point of 0.0.5

#### Control–control comparisons

Within the TD group there were no significant between-group differences in ED features over time.

## Discussion

We examined cross-sectional differences in ED features and comorbid psychopathology among adolescents with AN enrolled in our BRAVE study before and during the pandemic. In addition, we assessed the association between pandemic timing and 1-year clinical outcome. Peri-pandemic AN participants had fewer ED symptoms, were less dissatisfied with their body shape, and perceived less interpersonal insecurity compared to pre-pandemic AN participants. In addition, peri-pandemic AN participants fulfilled less DSM-IV criteria for comorbid disorders, in particular less anxiety disorders compared to pre-pandemic AN participants. Conversely, longitudinal analyses showed that the AN peri-pandemic group had a lower increase in BMI after 1-year follow-up compared to the AN pre-pandemic group. Among the reference group, TD girls, there were no significant differences in ED features and psychopathology between pre- and peri-pandemic TD girls.

### ED features

Interestingly, peri-pandemic AN participants reported less severe general ED symptomatology, perceived less concerns about diet, body weight and body shape compared to pre-pandemic AN participants. This appears to be in contrast with previous literature with a comparable case–case study design which showed either an increase in binge eating episodes [30] a trend towards more food restraint [29], or no significant differences in ED symptoms [28] among adolescents with an ED peri-pandemic versus pre-pandemic. The observed discrepancy could be explained by differences between the respective populations, e.g., age, severity of illness, duration of illness, specific time period during the pandemic, or differences in COVID-19 regulations imposed by different governments in response to the pandemic. The retrospective chart review by Spettigue et al. [29] studied a population with different types of EDs at high risk of medical complications, whereas we studied prospectively a patient group at different stages of illness. In addition, the current study collected data during a more extensive period during the pandemic (March 2020–October 2021), while previous studies [28, 29] collected data during a relatively short period during the pandemic (6–9 months). The study by Meneguzzo [30] conducted a study on a patient population that was relatively comparable to ours. However, their patient population had a slightly longer disease duration and their study was conducted in another country with different COVID-19-related restrictions at that specific time point. It is hypothesized that the interaction between imposed restrictions, coping strategies, and the presence of risk factors (i.e., fear of contagion, feeling of increased isolation) and resilience factors (more family cohesion, less social pressure) [53] contributed significantly to the symptom presentation of AN and may explain the differential findings. Two other studies conducted in both adolescent and adult inpatients with an ED [54, 55] using a similar case–case design also demonstrated higher levels of ED symptoms and psychopathology in the peri-pandemic ED cohort compared to the pre-pandemic ED cohort. As these studies did not differentiate between adolescents and adults, and were conducted in inpatients with different types of ED, they were not directly comparable to our study.

Another finding is that peri-pandemic AN participants were less insecure during social interactions compared to pre-pandemic AN participants. Possibly, reduced social interactions with peers might have a positive impact on social insecurity among adolescents with AN, since peer relationships could contribute to the disorder’s persistence [56]. Moreover, virtual social interactions, in which one sees only someone's face instead of their whole body, were predominant during the COVID-19 pandemic. This may be less confronting for participants with AN and potentially beneficial for their ED symptoms [56]. Additionally, during the COVID-19 pandemic AN participants were more in their home environment because of school closure and had more interactions with their parents, which has the potential to create a more protective environment and facilitate earlier detection of an ED [57, 58]. Strong parent–child relationships are linked to social competence [59], and the quality of friendships [60], potentially leading to a decrease in interpersonal insecurity.

BMI-SDS and physical exercise did not differ between the pre- and peri-pandemic AN group, which contradicts the findings of Datta et al. [28] where peri-pandemic AN participants had a lower BMI and increased over-exercising. Interestingly, our study reveals that peri-pandemic AN participants had less hand grip strength and a lower arm circumference compared to pre-pandemic AN participants. Although these findings lost significance after multiple-testing correction. In addition to BMI-SDS, arm circumference and hand grip strength are considered as markers of nutritional status [61–64]. It is speculated that arm circumference and hand grip strength may be more effective than BMI-SDS in detecting subtle differences, suggesting that peri-pandemic AN participants experienced poorer nutritional status compared to those included pre-pandemic. Furthermore, peri-pandemic AN participants had less weight gain after 1 year of follow-up compared to pre-pandemic AN participants. It is possible that decreased accessibility of health care facilities peri-pandemic [24] or the slightly higher body weight of the peri-pandemic group at baseline resulted in less catch-up in weight.

Peri-pandemic TD girls had lower perceptions of fear towards maturity compared to pre-pandemic TD girls. However, this effect ceased to be significant after multiple-testing correction. No other cross-sectional differences in ED symptoms were observed between the pre- and peri-pandemic TD groups. This finding is not in line with the increased prevalence of EDs within the general population during the pandemic [65, 66]. Our findings suggest that TD girls experienced less social pressure [67], and increased family cohesion during the peri-pandemic period, potentially protecting against the emergence of psychopathology [68].

### Comorbid psychopathology

Peri-pandemic AN participants fulfilled fewer DSM-IV classifications compared to pre-pandemic AN participants, primarily due to fewer anxiety disorder classifications. Remarkably, psychopathology symptoms did not significantly differ between pre- and peri-pandemic AN participants, consistent with Springall et al. findings [27]. One potential explanation for the divergence in psychopathology classifications and symptoms is that peri-pandemic AN participants may have experienced fewer anxiety-inducing situations, such as reduced physical gatherings, and less crowding in public. As a result, their daily lives may have been less impacted by anxious feelings, resulting in fewer DSM-IV anxiety disorder classifications.

### Strengths and limits

The study's strengths involve the application of objective measures alongside self-report data, as well as the use of a longitudinal design and a matched reference group in terms of age, gender, and education. Additionally, the emergence of the COVID-19 pandemic amidst our data collection allowed for analysis of the pandemic’s impact on illness severity upon presentation and disease progression in adolescent AN.

There may be limitations due to our extensive test battery, which could result in a possible absence of severe AN cases, as participants must be psychically and mentally capable of taking part in our study The number of measures and statistical analyses performed increased the probability of Type I errors, therefore a correction for multiple testing (i.e., Benjamini–Hochberg adjustment) was applied to reduce the risk of false-positive results. Another limitation of this study is the relatively high drop-out rate during follow-up in the AN group compared to the TD group. Although this drop-out rate is consistent with those reported in other longitudinal studies on AN [69], it may have influenced the results. It is unclear whether the patients who dropped out had a more favorable or more severe disease course compared to those with available follow-up data. We anticipate that this effect will be insignificant since we found no differences in sociodemographic and clinical variables between AN participants with and without follow-up data at the time of inclusion. Additionally, since our study only consisted of participants with AN, we cannot draw conclusions for other EDs. Lastly, there were slight differences in recruitment methods for AN participants between pre- and peri-pandemic periods, with fewer recruited through their healthcare providers during the latter. This could be attributed to the shift from in-person appointments to teleconsultations, and longer waiting lists of specialized services for eating disorders. The pandemic has resulted in greater reliance on social media advertising for recruitment. It is noteworthy that most participants recruited via social media received care through mental health institutions within our collaborative network. Furthermore, there were no significant differences in ED features, psychopathology, and duration of illness among participants enrolled through their healthcare provider and those recruited through social media during the peri-pandemic period, indicating minimal selection bias.

### Conclusion

In conclusion, this study shows that adolescents with AN enrolled peri-pandemic had fewer ED symptoms, were less dissatisfied with their body shape, perceived less interpersonal insecurity, and fulfilled fewer DSM-IV criteria for comorbid psychiatric disorders, compared to AN participants enrolled pre-pandemic. At the same time, there were no cross-sectional differences in ED features and psychopathology between pre-pandemic and peri-pandemic TD girls. The exploratory analyses showed that the peri-pandemic AN group gained less weight over time, compared to the pre-pandemic AN group, which was possibly due to less access to mental health services in the peri-pandemic period. Mental health services should be aware of a less favorable clinical course under stressful conditions such as the COVID-19 pandemic. Our findings are important for the clinical management of AN patients, by increasing awareness of the effects of the pandemic on ED features, psychopathology, and the course of the disease. Future studies are needed not only to replicate our findings, but also to investigate the mechanisms by which the COVID-19 pandemic affected the physical and mental health of adolescents with peri-pandemic diagnosed AN. Furthermore, our study indicates the need for careful interpretation of scientific results based on peri-pandemic conditions, as we have shown that pre- and peri-pandemic enrolled AN groups are not directly comparable. Therefore, we recommend studies that enrolled participants both during the pandemic and before or after the pandemic take into account the timing of the pandemic.

## What is already known on this subject?

There has been a rapid increase in ED presentations since the onset of the COVID-19 pandemic. Studies focusing on the impact of the COVID-19 pandemic on the behavior and mental health of individuals with AN have shown mixed results. While some studies found a worsening of ED symptoms and increased levels of psychopathology, others showed an improvement in ED symptoms possibly due to less peer pressure and more time for self-reflection during the pandemic period compared to before the pandemic. To date, there has been a great deal of heterogeneity in the methodological approaches used in previous studies, which makes comparability between studies difficult. In particular, age and disease stage may influence the severity of ED symptoms, comorbid psychopathology, and the way in which individuals perceive the pandemic [70]. To date, most studies have focused on adults with long-standing ED, while few studies have focused on young people with a short-lived illness. In the BRAVE study, we assessed a subset of people with first-onset AN at different stages of the disease who were physically and mentally able to participate in the study. Therefore, our findings cannot be extrapolated to all people with ED.

## What this study adds?

This study shows that adolescents with first-onset AN recruited during the COVID-19 pandemic had less severe ED symptoms and comorbidity at baseline than those recruited before the pandemic. However, AN participants recruited during the pandemic had less weight gain at 1-year follow-up. While most studies have not systematically collected data, our study comprehensively assessed AN participants in multiple domains during face-to-face appointments at two timepoints, allowing us to assess the clinical course over one year. Furthermore, we used an age-, gender-, and education-matched control group as a reference. Our results underline the importance of addressing the impact that the COVID-19 pandemic has had on AN patients, as well as improving patient management by highlighting the differences in clinical features between pre- and peri-pandemic first-onset AN patients. Moreover, our study contributes to the careful interpretation of research carried out in this area during the COVID-19 pandemic.

### Supplementary Information

Below is the link to the electronic supplementary material.

Supplementary file1 (DOCX 81 KB)

## Data Availability

Data access and requests for collaboration are welcome, and will be conducted in accordance with the European Union's General Data Protection Regulation (GDPR).
