# Supplementary material for: The implications of the COVID-19 pandemic on eating disorder features and comorbid psychopathology among adolescents with anorexia nervosa and matched controls: a comparative cohort design study
Source: Eat Weight Disord. 2024 Feb 12;29(1):13. doi: 10.1007/s40519-024-01640-0 (PMC10861646; doi:10.1007/s40519-024-01640-0)
Supplement: Supplementary file 1 — Supplementary file1 (DOCX 81 KB) [file 40519_2024_1640_MOESM1_ESM.docx]

SUPPLEMENTARY MATERIAL

**Supplemental table 1.** ED features and comorbid psychopathology of AN participants recruited via different methods peri-pandemic.

|  |  | Via health care provider (n=18) | Via social media (n=10) | Otherwise (n=2) |  |
| --- | --- | --- | --- | --- | --- |
|  | N | Mean (SD) | Mean (SD) | Mean (SD) | *P* value |
| **ED features** |  |  |  |  |  |
| BMI-SDS | 27 | -0.86 (1.28) | -1.52 (1.51) | -2.22 (0.37) | .275 |
| EDE total score | 30 | 2.68 (1.42) | 2.71 (1.66) | 4.06 (0.45) | .462 |
| **Comorbid psychopathology** |  |  |  |  |  |
| Depression | 28 | 19.00 (9.63) | 26.7 (12.48) | 33.50 (19.09) | .109 |
| Anxiety | 28 | 36.31 (20.54) | 47.70 (25.09) | 52.50 (19.09) | .359 |
| Compulsory thoughts | 28 | 3.94 (4.88) | 5.00 (6.60) | 11.00 (4.24) | .253 |
| Compulsory habits | 28 | 3.44 (4.69) | 5.10 (5.95) | 12.50 (4.95) | .082 |
| Autism (SRS-2) | 19 | 51.13 (7.82) | 54.75 (6.84) | - | .413 |
| Autism (SRS-A) | 4 | - | 70.25 (4.92) | 74.00 (7.07) | .478 |
| Number of comorbid DSM-IV classifications (MINI-interviews) | 29 | 0.71 (0.85) | 1.70 (1.42) | 1.00 (1.16) | .109 |

*BMI-SDS: body mass index – standard deviation score; ED: eating disorder; EDE: Eating Disorder Examination; SRS-2: Social Responsiveness Scale – Second edition. SRS-A: Social Responsiveness Scale – Adults.*

**Supplemental table 2.** Sociodemographics, eating disorder features, and psychopathology of AN participants and TD girls pre- and peri-pandemic.

|  |  | Pre-pandemic (n=86) | |  |  | Peri-pandemic (n=68) | |  |
| --- | --- | --- | --- | --- | --- | --- | --- | --- |
|  |  | AN participants (n=49) | TD girls (n=37) |  |  | AN participants (n=30) | TD girls (n=38) |  |
|  | *N* | Mean (SD) | Mean (SD) | *P* value | *N* | Mean (SD) | Mean (SD) | *P* value |
| **Sociodemographics** |  |  |  |  |  |  |  |  |
| Age (years) | 86 | 16.08 (2.06) | 17.29 (2.34) | .241 | 68 | 16.99 (2.41) | 17.19 (2.16) | .203 |
| Ethnicity (%)  Dutch  Western  Non-western | 82 | 97.8  2.2  0 | 97.2  2.8  0 | .861 |  | 93.1  3.4  3.4 | 97.4  0  2.6 | .417 |
| Educational level of mother (%)  Low  Middle  High | 79 | 15.9  40.9  43.2 | 2.9  42.9  54.3 | .158 | 64 | 10.3  20.7  69.0 | 8.6  22.9  68.6 | .987 |
| FSIQ-score | 86 | 106.67 (12.67) | 112.41 (12.72) | .528 | 67 | 113.34 (10.71) | 110.82 (10.67) | .791 |
| **ED features** |  |  |  |  |  |  |  |  |
| BMI-SDS | 81 | -1.29 (1.21) | 0.28 (0.83) | <.001* | 64 | -1.25 (1.40) | 0.61 (1.01) | <.001* |
| Hand grip strength, ‡  Arm circumference, ‡ | 56  55 | 24.49 (4.66)  231.80 (27.73) | 25.05 (6.40)  248.26 (15.96) | .946  .031* | 65  65 | 22.11 (4.87)  213.35 (25.16) | 24.67 (4.45)  252.43 (28.57) | .030  <.001* |
| Low intensity exercise (h/week),‡  High intensity exercise (h/week),‡ | 51  50 | 3.27 (3.98)  1.92 (2.34) | 2.79 (2.46)  4.09 (2.83) | .704  .477 | 63  64 | 5.40 (6.41)  1.96 (2.14) | 3.36 (2.26)  3.08 (3.32) | .343  .813 |
| EDE total score | 86 | 3.90 (0.93) | 0.33 (0.73) | <.001* | 68 | 2.78 (1.47) | 0.47 (0.48) | <.001* |
| EDI total score | 74 | 157.28 (40.64) | 41.50 (22.16) | <.001* | 65 | 146.50 (42.76) | 45.73 (32.03) | <.001* |
| BSQ total score | 74 | 143.46 (31.17) | 50.67 (16.98) | <.001* | 65 | 122.43 (34.11) | 57.51 (18.42) | <.001* |
| **Comorbid psychopathology** |  |  |  |  |  |  |  |  |
| Depression | 71 | 27.00 (9.39) | 4.53 (4.45) | <.001* | 65 | 22.79 (11.82) | 4.59 (5.06) | <.001* |
| Anxiety | 71 | 45.91 (21.07) | 21.41 (14.13) | <.001* | 65 | 41.54 (22.29) | 23.03 (15.76) | <.001* |
| Compulsory thoughts | 65 | 3.51 (5.99) | 0.03 (0.18) | .003* | 65 | 4.82 (5.63) | 0.84 (2.92) | .910 |
| Compulsory habits | 65 | 5.14 (6.67) | 0.63 (2.01) | .029* | 65 | 4.68 (5.52) | 0.51 (2.21) | .819 |
| Autism (SRS-2) | 16 | 56.71 (10.22) | 44.14 (6.33) | <.001* | 12 | 51.89 (7.59) | 48.19 (11.14) | <.001* |
| Autism (SRS-A) | 57 | 72.00 (1.73) | 68.09 (1.45) | <.001* | 45 | 71.50 (5.32) | 68.17 (1.17) | <.001* |

|  |  | Pre-pandemic (n=86) | |  |  | Peri-pandemic (n=68) | |  |
| --- | --- | --- | --- | --- | --- | --- | --- | --- |
|  |  | AN participants (n=49) | TD girls (n=37) |  |  | AN participants (n=30) | TD girls (n=38) |  |
|  | *N* | Percentage | Percentage | *P* value | N | Percentage | Percentage | *P* value |
| Comorbid psychopathology |  |  |  |  |  |  |  |  |
| Number of DSM-IV classifications (MINI-interviews) |  |  |  | <.001* |  |  |  | <.001* |
| 0 | 39 | 14.3 | 88.9 |  | 41 | 37.9 | 78.9 |  |
| 1 | 19 | 32.7 | 8.3 |  | 17 | 37.9 | 15.8 |  |
| 2 | 21 | 40.8 | 2.8 |  | 3 | 6.9 | 2.6 |  |
| 3 | 4 | 8.2 | 0 |  | 4 | 13.8 | 0 |  |
| 4 | 1 | 2.0 | 0 |  | 0 | 0 | 0 |  |
| 5 | 1 | 2.0 | 0 |  | 0 | 0 | 0 |  |
| Any mood disorder | 39 | 69.4 | 13.9 | <.001* | 20 | 51.7 | 13.2 | <.001* |
| Any anxiety disorder | 34 | 61.2 | 11.1 | <.001* | 9 | 17.2 | 10.5 | .425 |
| Any OCD | 10 | 20.4 | 0 | .004* | 8 | 24.1 | 2.6 | .007* |
| Any behavior disorder | 1 | 2.0 | 0 | .389 | 2 | 6.9 | 0 | .100 |
| Any ADHD | 5 | 4.1 | 2.8 | .748 | 4 | 6.9 | 5.3 | .780 |
| Any ASD | 0 | 0 | 0 | - | 0 | 0 | 0 | - |

*AN: anorexia nervosa; TD: typically developing; ED: eating disorder; ^A^Low: primary education and lower general secondary education; Middle: higher general secondary education; High: higher vocational secondary education and higher academic education; FSIQ-4: Full Scale Intelligence Quotient; BMI-SDS: body mass index – standard deviation score; EDE: Eating Disorder Examination; EDI: Eating Disorder Inventory; BSQ: Body Shape Questionnaire; BDI-II: Beck Depression Inventory – Second edition; SCARED: Screening for Child Anxiety Related Emotional Disorders; Y-BOCS: Yale-Brown Obsessive-Compulsive Scales; SRS-2: Social Responsiveness Scale – Second edition. SRS-A: Social Responsiveness Scale – Adults.* ‡ *Data available from a limited number of participants, because these measures were no part of the study protocol until 24/10/2017. *: significant p-value after correction for multiple testing using FDR with a cut-off point of .05.*


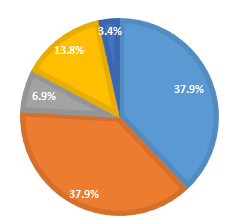
*
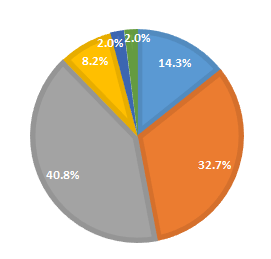
***Supplemental Fig. 1 -** Number of DSM-IV classifications (MINI-interviews) of AN participants pre- and peri-pande*
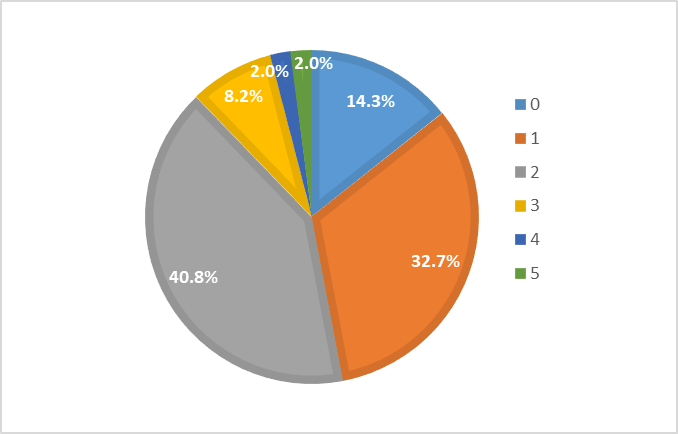
*mic

2b. Peri-pandemic (n=30)

2a. Pre-pandemic (n=49)

**Supplemental table 3.** DSM-IV anxiety subclassifications among AN participants pre- and peri-pandemic.

|  | Pre-pandemic | Peri-pandemic |
| --- | --- | --- |
| Number of DSM-IV classifications |  | |
| **MINI-KID** | AN participants (n=40) | AN participants (n=20) |
| Panic disorder | 4 | 0 |
| Agoraphobia | 8 | 1 |
| Separation anxiety disorder | 4 | 0 |
| Social anxiety disorder | 11 | 0 |
| Post-traumatic stress disorder | 0 | 1 |
| Generalized anxiety disorder | 12 | 2 |
| Specific Phobia | 9 | 0 |
| **MINI-PLUS** | AN participants (n=9) | AN participants (n=9) |
| Panic disorder with agoraphobia | 1 | 1 |
| Panic disorder without agoraphobia | 0 | 0 |
| Agoraphobia with a panic attacks in the history | 0 | 0 |
| Agoraphobia without a panic disorder in the history | 1 | 1 |
| Social anxiety disorder | 2 | 2 |
| Post-traumatic stress disorder | 2 | 2 |
| Generalized anxiety disorder | 3 | 1 |
| Specific phobia | 2 | 0 |
| Hypochondria | 0 | 0 |

*AN: anorexia nervosa; Mini International Neuropsychiatric Interview (MINI).*
